# Supplementary material for: The evolution, distribution and diversity of endogenous circoviral elements in vertebrate genomes
Source: Virus Res. 2019 Mar;262:15–23. doi: 10.1016/j.virusres.2018.03.014 (PMC6372831; doi:10.1016/j.virusres.2018.03.014)
Supplement: Supplementary file 1 [file mmc1.docx]

**Figure S1. (a)** A maximum likelihood phylogeny showing estimated evolutionary relationships between eCVe Rep amino acid sequences recovered from the genome of the inshore hagfish (*Eptatretus burgeri*). **(b)** A maximum likelihood phylogeny showing estimated evolutionary relationships between eCVe and circoviruses, and including short matches. In both panels, the scale bars show evolutionary distance in substitutions per site. Bootstrap support was assessed using 1000 bootstrap replicates. Asterisks indicate nodes with bootstrap >75%.

**Figure S2**. A multiple sequence alignment of virtually translated CVe recovered from the genome of snakes (suborder Serpentes). Sequence IDs correspond to the accession numbers of contigs in which CVe occur.
