# Supplementary figures and images for: The evolution, distribution and diversity of endogenous circoviral elements in vertebrate genomes
Source: Virus Res. 2019 Mar;262:15–23. doi: 10.1016/j.virusres.2018.03.014 (PMC6372831; doi:10.1016/j.virusres.2018.03.014)

a)

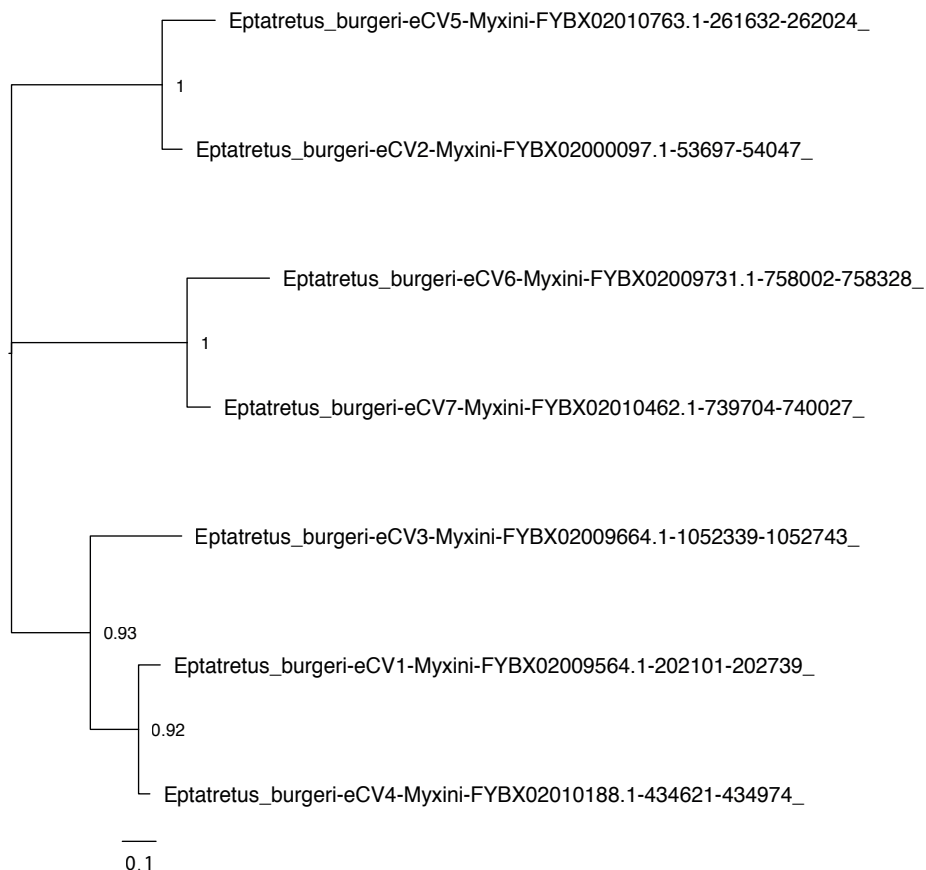

b)

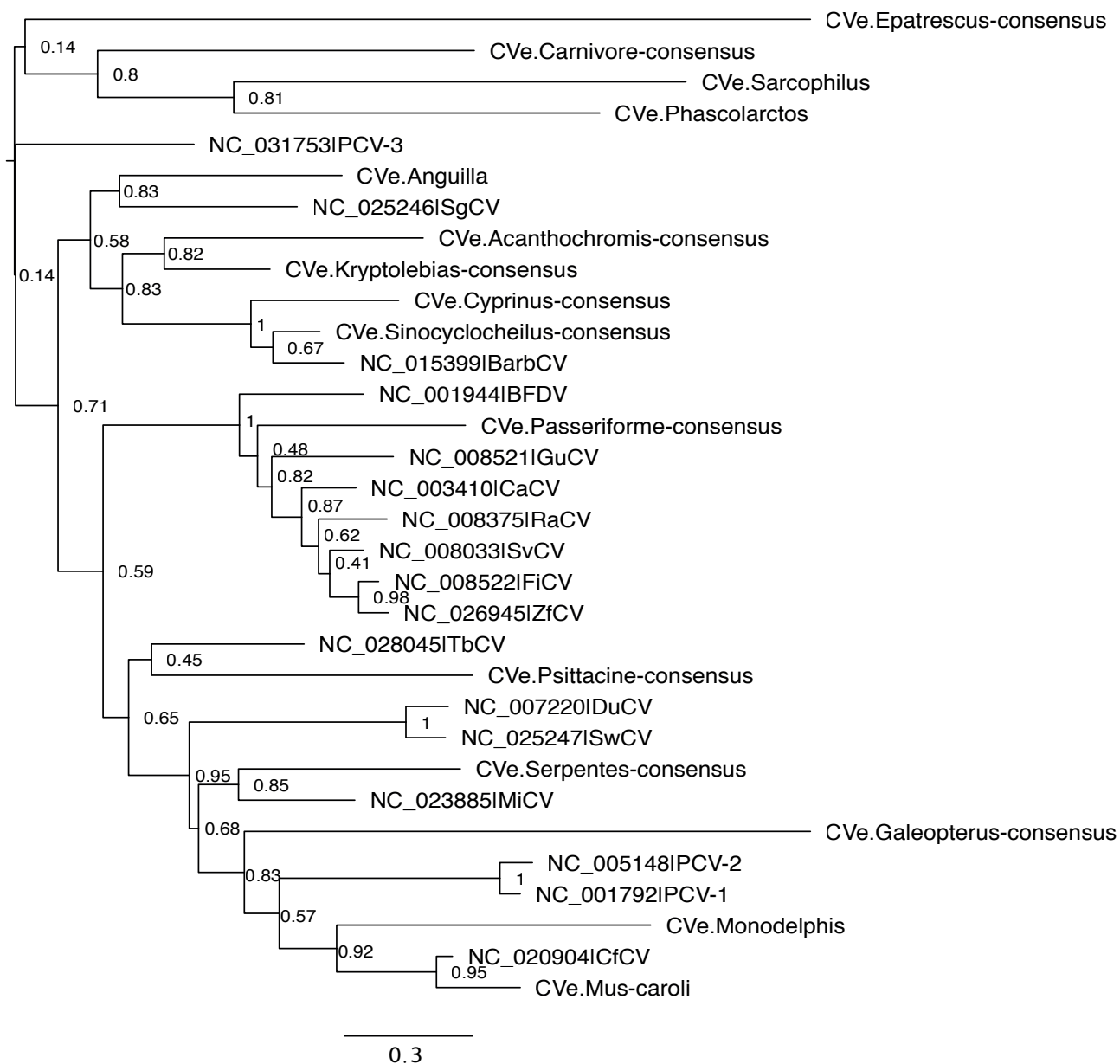

Supplement: Supplementary file 2 [file mmc2.pdf]
