# Supplementary material for: The evolution, distribution and diversity of endogenous circoviral elements in vertebrate genomes
Source: Virus Res. 2019 Mar;262:15–23. doi: 10.1016/j.virusres.2018.03.014 (PMC6372831; doi:10.1016/j.virusres.2018.03.014)
Supplement: Supplementary file 3 [file mmc3.pdf]

Figure S2.

```
LVCR01008418  35550  IPAIENMQLAGHPCKRYVFTINNPSEEDYDAVKDFITPENCVYAIVGEEQVSTLHLQGFV  35729
JPMF01009165  2134   IPAIENMQLAGHPCKRYVFTINNPSEEDYDAVKDFITPENCVYAIVGEEQVSTLHLQGFV  2313
LVCR01005637  76800     IETMQLAGHPCKRYVFTINNPSEEDYTAVKDFITPENCVYAIVGEEQVSTLHLQGFV  76630
JPMF01099474  4867      IKTMQLAGHPCK*YVFTINNPSEEDYTAVKDFITPENCVYAIVGEEQVSTLHLQGFV  5037
JPMF01327854  107                                                         LASFV  121
JPMF01327854  21                               KDYTAVKDFIIPENCVYAIVGEEQVSTLHLQALL  122
LVCR01018124  48796                         SEEDYNAVKDFITPENCVYAIVGEEQVCVYAIVG    48897
JPMF01003018  2944                          SKEDYNAVKDFITPKNCVYAIVGEEQVCVYTIVG    2843

LVCR01008418  35730  NLKKKMRFNPFKAAIGGRAHVEKAKRTDLDNKRYCSKQKVYLEVGEPSAQGKRLDLKEAI  35909
JPMF01009165  2314   NLKKKMRFNPFKAAIGGRAHVEKAKGTDLDNKRYCSKQKVYLEVAEPSAQGKRLDLKEAI  2493
JPMF01024207  6477                                                  TAQEKRSDLKEAI  6515
LVCR01005637  76629  NLKKKMRFNPFKAAV                                               76585
JPMF01099474  5038   NLKKKMRFNPFKAAV                                               5082
JPMF01327854  122    NLKKKMRFNPFKAAIGGRAHIEQAKGTNLDNKTYCSKQKLVYQEG                 256
AEQU02103080  78       QEKEEFIVVKEMLGGRAHLEKTKGTDLDNQTYCCKGGDFIEISESSKLGKRNDLDNAV  251

LVCR01008418  35910  ALLNSGSSMTELAQAYPSVYIRYGRGL-----KDYVITAQLTRMRDFKTDVHVITGIPGC  36074
JPMF01009165  2494   ALLNSGSSMTELAQAYPSVYIRYGRGL-----KDYVITAQLARMRDFKTDVHVITGIPGC  2658
JPMF01192303  58                                           AQLTKMRDFKTEVHVIMGIPRC  123
LVCR01015089  21904                                          AQLAKMRDFKTEVHVITGIPGC  21839
JPMF01024207  6516   ALLNSGSSMTELARAYPSVYIRYGMGL-----IDYVITAQLAKMRDFKTDVHVITGIPGC  6680
AEQU02103080  252    EQLH                                                          263
AEQU02103080  266           NLTEVAHEYPATFIKYGHALVCTYVRTYVI                         355

LVCR01008418  36075  GKSKFCSELDGDKYWKPRGKWWDGYTGQEI--VILDD-FYGWL-P----FDELLRICDRY  36230
JPMF01009165  2659   GKSKFYSELDGDKYWKPRGKWWDGYTGQEI--VILDD-FYGWL-P----FDKLLRICDRY  2814
JPMF01192303  124    GKSKFCNELEGEKYWKPRGKWWDGYTGQEI--VILDD-YYGWL-P----FDELLRLCDRY  279
LVCR01015089  21838  GKSKFCNELEGEKYWKPRGKWWDGYTGQEI--VILDD-YYGWL-P----FDELLRLCDRY  21683
JPMF01024207  6681   GKSKFCSELDGDKYWKPHGKWWDGYTGQEI--VILDD-FYGWI-P----FDELLRICDRY  6836
LVCR01091779  7273                                                       LLNLCLRV  7296
BCNE02020765  4847                                      DD-FYGWLLP----FDELLRVCDRY  4906
BCNE02020767  1611                                      DD-FYGWLLP----FDELLRVCDRY  1670
BCNE02075733  4046                        WDGYFGQKV--AITDDFFYGWI-PRMNCWD*GINI---H  4144
LVCR01061408  7929                        WDGYSGQKV--AITDDFFYGLD-T----TDEL*GLTDKY  8024
AZIM01004063  27509                       WDGYSGQKVGNKYMIF-LKKWLDT----TDESLGLMDKY  27610

LVCR01008418  36231  PYRVET---KDGTMEFVSKCIYITSNKPVKEWYDEDIHT---ET                  36344
JPMF01009165  2815   PYRVET---KGGTMEFVSKCIYITSKQ                                   2886
JPMF01223472  1153     RVET---KGGTVEFVSKSIYITSNKPVKEWYSEEYCL---EALFRRITSYKIMRKGEG  998
JPMF01192303  280    PYRVET---KGGTVEFVSKTIYITSNKPVKEWYNEEYCL---EALFRRITSYKIMRKGEG  441
LVCR01015089  21682  PYRVET---KGGTIEFVSKTIYITSNKPVKEWYNEEYCL---EALFRRITSYKIMRKGEG  21521
JPMF01024207  6837   SYRVET---KGGTMEFVSKSI                                         6890
LVCR01091779  7297   YISLQTNL*KSGIVRSIVLRHCLEESLHTK**EKEKGCLIKCQLYFQSIVKYIHMEK---  7467
LVCR01091779  7251     RVET---KGGTVEFVSKSIYITSNKPVKEWYSEEYCL---EALFRRITSYKIMRKGEG  7406
BCNE02045840  57610                                              IRRITSYKIMRKGEG  57566
BCNE02020765  4908   PTELRP---RGATMEFVSKKIYITSNKPVKEWYGEECCM---QALFRRITTYQIMTTGEG  5069
BCNE02020765  4907   PYRVET---KGGN                                                 4936
BCNE02020767  1672   PTELRP---RGATMEFVSTKIYITSNKPVKEWYGEECCM---QALFRRITTYQIM-TTEG  1830
BCNE02020767  1671   PYRVET---KGGN                                                 1700
BCNE02075733  4145   LTKWQV---KGSFQEFVSKKI                                         4198
LVCR01061408  8025   P                                                             8027
AZIM01004063  27611  PPKWQV---QGSFQEFVSKTINCPTN                                    27679

LVCR01008418  35113                                              TFWDIHTETKTPVE-  35072
LVCR01008418  36327                                                 DIHTETKTPVE-  36359
JPMF01009165  2909                                                 EDIHTETKTPVE-  2944
JPMF01009165  1715                                               TFWDIHTETKTPVE-  1674
JPMF01223472  997    MSDKMPTLFPINC*VHTHGKNKFSELNIVCDFY*P*VSNWLGGCETFRNIHAETKTPVEY  818
JPMF01192303  442    MSDKIPTLFPINC*ACV--HGKINSLN*T-*YVIL*VLYWLGSTETFWDIHSSAQ       597
LVCR01015089  21520  MSDKIP                                                        21503
LVCR01015089  21464                     KNKFSELNIVCYLY*P*VLYWLGSTETFWDIHSSAQ       21357
LVCR01091779  7468   ----------------------INSLNIVCDFY*P*VSNWLGGCETFRNIHAETKTPVEY  7581
LVCR01091779  7407   MSDKMPTLFPINC*VHTHGKNKFSEHSM                                  7490
BCNE02045840  57527  LSTSTW-------------KNKLSELNIVCEFN*PAVLYRFLQHSALRYIYTESQTPVD-  57390
BCNE02045840  57565  MSDKVP                                                        57548
JPMF01223473  3243                      KNKFSELNIVCDFYKP*VFYWLGSSETLWNIHTESKPPVEH  3121
BCNE02020765  5070   LKDKMP                                                        5087
BCNE02020767  1831   LKDKMP                                                        1848
JPMF01156588  204                           ARLNIVCYLY*P*VLYWLGSTEAF*NIHAESKSPMEH  314
BCNE02035370  8539                                                       ESQAPV*H  8562
JTLQ01205518  1347                      KYKFSELQVVCYFYIP*IGYIIS-----RYIHRQT*PIMG*  1454
```

```
LVCR01008418  36448                                 IIN*LGFGGEVS*VVS*EYPVVGS*LVHE  36534
LVCR01008418  35071  -NLVIIAVEPELVVFAVWLEED*ARGGGGGCIINHLEFG                      34958
LVCR01008418  36360  -NLVIIAVEPELVVFAVWLEED*TRGW                                  36437
JPMF01009165  3033                                  IIN*LGFGGEVS*VVS*EYPVVGS*LVHE  3119
JPMF01009165  2945   -NLVIIAVEPELVVFAVWLEEN*TR                                    3016
JPMF01009165  1673   -NLVIIAVEPELVVFAVWLEE------------D*VWGGG                     1593
JPMF01223472  817    IS--IFGIEPKLIIFIVWLEEC*FLDCCCALDIRELWFGCEVS*VVSVWDPVVC**SIHK  644
LVCR01091779  7582   IS--IFGIEPKLIIFIVWLEEC*FLDCCCALDIRELWFGCEVS*VVSVWDPVVC**SIHK  7755
BCNE02045840  57389  -NVVIIGIDPHLVIFIVGLEK--QRPLWCGFVSNQLWFWGEIS*VVSVEYPVVGC*SVHK  57219
JPMF01223473  3120   --GVIITI*P*LIIFIIWLEEEGPRG--CGFVLDQLGIGGEVS*VVSVGDPIVGC*PVLK  2953
BCNE02032238  3796    TVEKVGIEPKLVVF*IWLEE--HRSSWCHGVIDQLGFGGEVS*VWSGKDPVVGCGSVHE  3626
JPMF01333966  283                                   IIN*LGFGGEVS*VVS*EYPVVGS*LVHE  197
JPMF01156588  315    --GVIITIKPQLIIFIIWLEEEGPRG--CGLVCYDLGFGG                     422
BCNE02035370  8563   --GVIIGVEPELVVLAVWLEEH*SR--WGDTISYYLWFWGEVS*VISVEDPIMSCRSIHE  8730
JTLQ01205518  1455   I--ISFRI*PELIIFVIWLKP--FTSWW                                 1526
```

```
LVCR01008418  36535  GVLRG*SGPWGW*VSIKINNSGSSGYFFM*VKKGGQKFNFDLSDSIVIP  36681
JPMF01009165  3120   GVLRG*SGPWGW*VSIKINNSGSSGCFFM*VKKGGQKFNFDLSDSIVIP  3266
JPMF01223472  643    WVICCWGGGGCWNIAIKINNSGSS*VFFL*AQECWEEFNFELSDSIVIP  497
LVCR01091779  7756   WVICCWGGGGCWNIAIKINNSDSS*VFFL*AQECWEEFNFDLSDSIVIP  7902
BCNE02045840  57218  RVIWCHGCPWCW*VSIQINDSGAPRGFFM                      57132
JPMF01223473  2952   RVIWCSCCSRLW*VAIKINNSGSSGIFFL*AKKCGKKFNFHFSDPTVIP  2806
BCNE02032238  3625   WVFR*KGAPW*W*VAFKVNYCCSS*GFFM*EQVSWEEFNFDLSGSVIVP  3479
JPMF01333966  196    GVLRG*SGPWGW*VSIKINNSGSSGCFFMGVKKGGQKFHFDLSDSIVIP  50
BCNE02035370  8731   WIFSESSAPWCW*VAIKINNSGSS                           8802
```
