# Supplementary material for: The evolution, distribution and diversity of endogenous circoviral elements in vertebrate genomes
Source: Virus Res. 2019 Mar;262:15–23. doi: 10.1016/j.virusres.2018.03.014 (PMC6372831; doi:10.1016/j.virusres.2018.03.014)
Supplement: Supplementary file 5 [file mmc5.docx]

**Table S2. Circoviruses reference sequences**

| **sequence-ID** | **name** | **full_name** | **clade** |
| --- | --- | --- | --- |
| NC_001944 | BFDV | Beak and feather disease virus | Avian-1 |
| NC_003410 | CaCV | Canary circovirus | Avian-1 |
| NC_008033 | SvCV | Starling circovirus | Avian-1 |
| NC_008375 | RaCV | Raven circovirus | Avian-1 |
| NC_008521 | GuCV | Gull circovirus | Avian-1 |
| NC_008522 | FiCV | Finch circovirus | Avian-1 |
| NC_026945 | ZfCV | Zebra finch circovirus | Avian-1 |
| NC_007220 | DuCV | Duck circovirus | Avian-2 |
| NC_025247 | SwCV | Swan circovirus | Avian-2 |
| NC_015399 | BarbCV | Barbel circovirus | Fish-1 |
| NC_025246 | SgCV | Wels catfish circovirus | Fish-2 |
| NC_001792 | PCV-1 | Porcine circovirus 1 | Mammal-1 |
| NC_005148 | PCV-2 | Porcine circovirus 2 | Mammal-1 |
| NC_020904 | CfCV | Canine circovirus 1 | Mammal-1 |
| NC_023885 | MiCV | Mink circovirus | Mammal-1 |
| NC_031753 | PCV-3 | Porcine circovirus 3 | Mammal-2 |
| NC_028045 | TbCV | Mexican free-tailed bat circovirus | Mammal-2 |
